# Attitudes toward own aging and cognition among individuals living with and without dementia: findings from the IDEAL programme and the PROTECT study

**DOI:** 10.1186/s12877-022-03336-5

**Published:** 2022-08-04

**Authors:** Serena Sabatini, Anthony Martyr, Obioha C. Ukoumunne, Clive Ballard, Rachel Collins, Claire Pentecost, Jennifer M. Rusted, Catherine Quinn, Kaarin J. Anstey, Sarang Kim, Anne Corbett, Helen Brooker, Linda Clare

**Affiliations:** 1 Centre for Research in Ageing and Cognitive Health, College of Medicine and Health, South Cloisters, St Luke’s Campus, University of Exeter, Exeter, EX1 2LU UK; 2 NIHR ARC South West Peninsula (PenARC), University of Exeter, Exeter, United Kingdom; 3 School of Psychology, University of Sussex, Brighton, UK; 4 The Centre for Applied Dementia Studies, University of Bradford, Bradford, UK; 5 Ageing Futures Institute, University of New South Wales, Sydney, and Neuroscience Research Australia, Sydney, Australia; 6 Wicking Dementia Research & Education Centre, University of Tasmania, Hobart, Australia

**Keywords:** Subjective aging, Cognitive decline, Views of aging, Parkinson’s disease dementia, Dementia with Lewy bodies, Dementia subtypes, Cognitive subdomains, Dementia prevention, Alzheimer’s disease, Depression

## Abstract

**Background:**

It is unclear whether people with dementia (PwD) have more negative attitudes toward own aging (ATOA) than people without dementia and what factors influence ATOA among PwD. We investigated whether PwD have more negative ATOA than individuals without dementia and whether cognition and dementia subtype are associated with ATOA in PwD.

**Methods:**

Data from the IDEAL and PROTECT studies were used to compare ATOA between 1502 PwD (mean (SD) age = 76.3 (8.5)) and 6377 individuals without dementia (mean (SD) age = 66.1 (7.1)). Linear regressions and ANOVA were used.

**Results:**

PwD reported slightly more negative ATOA than people without dementia; this relationship disappeared after controlling for depression and self-rated health. In PwD more positive ATOA showed negligible associations with better general cognition, memory performance, verbal fluency, and visuospatial ability. However, after adjusting for covariates only better visuospatial ability predicted more positive ATOA. Additional analyses showed that before and after controlling for covariates, individuals with poorer self-reported visual acuity have more negative ATOA. Amongst dementia subtypes, people with Parkinson’s disease dementia and dementia with Lewy bodies reported most negative ATOA.

**Conclusions:**

ATOA between PwD and people without dementia do not differ. ATOA in PwD appear to be affected not by cognitive impairment but by other characteristics that vary across dementia subtypes. Among PwD, those with Parkinson’s disease dementia and dementia with Lewy bodies may have higher risk of experiencing negative ATOA due to the motor and visual impairments that they experience.

**Supplementary Information:**

The online version contains supplementary material available at 10.1186/s12877-022-03336-5.

## Background

As the population of older people increases globally [1], there is growing interest in subjective aging and how this relates to cognition and both physical and mental health. Attitudes toward own aging (ATOA) capture individuals’ evaluations of change happening in their lives as they age [2]. ATOA are influenced by societal and cultural beliefs about aging [2] with pervasive negative stereotypes portraying older adults as cognitively inferior to younger individuals [3]. Increasing age can lead to decline in most cognitive abilities including a decrease in processing speed, attention, memory, visuospatial abilities, executive functions, and in some aspects of language (e.g. word finding), thus confirming these negative stereotypes [4].

Among middle-aged and older individuals cognitive decline often leads to negative self-perceptions of aging and cognitive ability [5, 6]. Those with more negative ATOA are at higher risk of cognitive decline [5, 7, 8] and of developing Alzheimer’s-like neuropathology [7–10]. The negative impact of ATOA on cognition may be due to individuals with negative ATOA being less engaged in preventative behaviors [11] related to better maintenance of cognitive health [12]. Therefore, negative ATOA may increase the risk of developing dementia in later life. Alternatively, impaired cognition may confirm and increase the salience of negative ATOA. People with dementia (PwD) experience a significant and progressive decline in multiple cognitive domains, including memory and executive functioning, that may interfere with the ability to conduct everyday activities [13–15]. Moreover, PwD often have poor physical and mental health [16, 17] which may negatively impact on the maintenance of positive ATOA. Finally, the progress of dementia may restrict physical and social activities that may directly impact quality of life [18] and ATOA. Therefore, due to having cognitive and functional impairments PwD may have more negative ATOA than people without dementia.

Only two cross-sectional studies have compared ATOA in people living with and without dementia [19, 20]. Both studies found that PwD were more likely to strongly express negative ATOA compared to those without dementia; however, the difference in levels of ATOA between PwD and people without dementia was small in both studies. Neither study adjusted for the effects of potentially important health-related covariates, such as poor mental and/or physical health [16, 17]. Such factors predict more negative ATOA in PwD and people without dementia [21]. In contrast, one study reported more positive ATOA in people with “suspected dementia” compared to individuals without dementia [22]. However, the reliability of this study is limited as participants in the “suspected dementia” group had not received a formal diagnosis of dementia. Research comparing levels of ATOA among PwD and people without dementia is, therefore, limited and equivocal.

Among PwD those with more negative ATOA have higher levels of depression, pain, functional impairment, and self-reported memory problems [19, 20] compared to those with more positive ATOA. Among PwD there is little evidence about the association of ATOA with cognition. One small-scale study which considered global cognition rather than specific cognitive abilities found no association between cognition and ATOA [20]. In previous ATOA studies PwD have been treated as a homogeneous group [19, 20] but different dementia diagnostic groups may show differences in ATOA due to disparate symptomatology [14]. Small sample sizes [19, 20] have hitherto prevented investigating diagnostic differences in ATOA.

Exploring whether PwD have more negative ATOA than people without dementia is important as negative ATOA may decrease individuals’ capability to live well with dementia; that is, the ability to experience the best health state that encompasses all dimensions of physical, mental, and social well-being [23]. PwD with more negative ATOA score more poorly on indicators of capability to live well (e.g. quality of life) compared to those with more positive ATOA [19, 20, 24]. Therefore we investigated in two large cohorts whether PwD have more negative ATOA than people without dementia, controlling for mental and physical health and functional ability. In PwD we explored whether ATOA are associated with cognition and cognitive subdomains and whether levels of ATOA differ across dementia subtypes.

## Methods

### Study design and participants

Cross-sectional data from two cohort studies was used. Data for PwD were collected as part of the Improving the experience of Dementia and Enhancing Active Life (IDEAL) programme baseline assessment between 2014 and 2016 [25, 26]. Analyses were conducted on version 4.5 of the dataset. PwD were recruited through a network of 29 National Health Service (NHS) Clinical Research Network (CRN) sites in Great Britain. Participants needed to have a dementia diagnosis of any type, a Mini-Mental State Examination (MMSE) score ≥ 15 (corresponding to mild-to-moderate dementia), and to be living in their own homes. Potential participants were excluded if they had a co-morbid terminal illness, were unable to provide informed consent, or there was any known potential for home visits to pose a significant risk to researchers. Further information about recruitment in IDEAL is reported in the published protocol [25]. IDEAL was approved by the Wales 5 Research Ethics Committee (reference: 13/WA/0405) and the Ethics Committee of the School of Psychology, Bangor University (reference: 2014–11,684) and is registered with the UK CRN (registration number: 16593).

Data for people without dementia were collected through the Platform for Research Online to investigate Genetics and Cognition in Aging (PROTECT, https://www.protectstudy.org.uk) study between 1st January and 31st March 2019. PROTECT participants were UK residents, English speakers, aged ≥50 years, had access to the internet, and did not have a clinical diagnosis of dementia. The PROTECT study was advertised through national publicity and through existing cohorts of older adults (Exeter 10,000 https://exetercrfnihr.org/about/exeter-10000/; Join Dementia Research https://www.joindementiaresearch.nihr.ac.uk/; and Brains for Dementia Research https://bdr.alzheimersresearchuk.org) [27, 28]. PROTECT was approved by the London Bridge NHS Research Ethics Committee and Health Research Authority (reference: 13/LO/1578) and permission to conduct data analyses was obtained through the ethics committee at the University of Exeter, School of Psychology (reference: eCLESPsy000603).

### Instruments

#### Attitude toward own aging

The Attitude Toward Own Aging (ATOA) questionnaire, taken from the Philadelphia Geriatric Center Morale Scale [2], was used to assess ATOA in both studies. Total scores ranged from zero (most negative response in all five answers) to five (most positive response in all answers). To maximize the use of data a pro-rata score was computed when a response to one of the five questions was missing [29].

#### Measures administered to PwD

Included measures were selected from the wider IDEAL dataset. Except where noted all measures used in the present study were administered to PwD by a researcher, including ATOA, and reflect information self-reported by PwD.

The Addenbrooke’s Cognitive Examination-III (ACE-III [30];) was used to measure cognition. Five subscales (attention; 0–18, memory; 0–26, verbal fluency; 0–14, language; 0–26, visuospatial ability; 0–16) are used, forming a total score for general cognition (0–100). Higher scores indicate better cognitive functioning. The ten-item Geriatric Depression Scale [31] was used to assess depression. Self-rated health was assessed with a single-item question [32] that asked participants to rate their own health on a six-point scale (excellent to very poor). To assess functional ability an amended 11-item Functional Activities Questionnaire (FAQ; 33) that includes an additional question concerning appropriate telephone use was used [13]. Higher scores indicate poorer functional ability. The Charlson Comorbidity Index [33, 34] was used to enumerate co-morbid conditions and was administered as a joint interview between the PwD and family caregiver where available. Participants indicated whether they had any of the 23 conditions. As dementia is one of the 23 conditions total scores ranged from 1 to 23. Higher scores indicate greater level of co-morbidity [17]. Self-reported visual acuity was assessed with a single-item question asking participants to rate their eyesight on a five-point scale (excellent to poor) [35]. Information regarding the subtype of dementia was obtained from participants medical records and classified in seven groups: Alzheimer’s disease, vascular dementia, mixed Alzheimer’s disease/vascular dementia, frontotemporal dementia, Parkinson’s disease dementia, dementia with Lewy bodies, and unspecified/other. Education was assessed as a dichotomous variable; below university or university. Employment was assessed as a categorical variable: Paid employment, Retired, Unemployed/doing voluntary (unpaid) work.

#### Measures administered to people without dementia

Included measures were selected from the wider PROTECT dataset. All measures were self-completed through the PROTECT online platform, including ATOA. Depression was assessed using the nine-item Patient Health Questionnaire (PHQ-9; 37). Self-rated health was assessed with a single-item question that asked participants to rate their own health on a four-point scale (excellent to poor) [36]. To assess functional ability a modified version of the Instrumental Activities of Daily Living Scale (IADL [37];) was used; two of the original eight items (assessing laundry and housekeeping) were combined.

Education was assessed as a dichotomous variable (below university or univeristy). Employment was assessed as a categorical variable: Paid employment, Retired, Unemployed/doing voluntary (unpaid) work. Cognitive functioning was measured with the PROTECT Cognitive Test Battery [28] which includes four tests (Grammatical Reasoning, Digit Span, Self-ordered Search, Paired Associate Learning).

### Harmonization

To compare the two sets of data some measures were harmonized. Depression scores were converted into a dichotomous variable (depressed; 4–10 on the Geriatric Depression Scale and 4–27 on the PHQ-9; not depressed; 0–4 on the Geriatric Depression Scale and 0–4 on the PHQ-9) [24, 38]. To match the responses in PROTECT the six-item self-rated health measure used in IDEAL combined *excellent* and *very good* to form the excellent rating while *very poor* and *poor* were collapsed into poor. Functional ability scores were converted into a dichotomous variable (dependent: 5–33 on the FAQ and 1–14 on the IADL; independent: 0–5 on the FAQ and 0 on the IADL) [39, 40].

### Data analysis

The two samples were combined and a dichotomous variable was created to classify whether people had dementia [1] or not (0). Linear regressions were conducted to explore whether dementia explained variability in levels of ATOA. To explore whether cognition explained variability in ATOA in PwD we conducted linear regressions. Analysis of variance and linear regressions explored whether levels of ATOA vary across dementia subtypes. To explore whether self-reported visual acuity explained variability in ATOA in PwD we conducted linear regressions. For each analysis both unadjusted models and adjusted models controlling for age, sex, education, employment, depression, self-rated health, and functional ability were conducted. In analyses with PwD, co-morbidity and self-reported visual acuity were included as covariates. Standardized regression coefficients were used to indicate effect size in regression models; ≤ 0.09 were considered negligible, 0.10–0.29 small, 0.30–0.49 moderate, ≥ 0.50 large [41]. Eta-squared and partial eta-squared (η^2^/pη^2^) were used to indicate effect size in ANOVA; 0.01–0.05 were considered small, 0.06–0.13 moderate, ≥ 0.14 large [41].

## Results

### IDEAL sample characteristics

Of the 1540 participants who took part in IDEAL, 38 participants were excluded from the analyses as they did not complete the ATOA scale. A further 305 had one missing ATOA item so a pro-rata score was computed and used in analyses. The sample included in this study comprised 1502 PwD, of whom just over half were diagnosed with Alzheimer’s disease, just over half were male, mean age was 76.3 years (range: 43–98), and the majority were white (99.7%). Further characteristics of the study sample are reported in Table 1.

**Table 1:** Descriptive statistics of demographic variables and main study variables for the IDEAL study sample

Characteristic	Sample who took part in the IDEAL study at baseline (*n* = 1502)	Missing	Sample who took part in the PROTECT study in 2019 (*n* = 6053)	Missing
Age in years, Mean (SD)	76.3 (8.5)	0	66.1 (7.1)	0
Sex (Women; n (%))	657 (43.7)	0	4612 (76.2)	0
Education (n (%))		34		0
Below university	1176 (80.1)		1491 (24.6)	
University	292 (19.9)		4562 (75.4)	
Current employment (Yes; n (%))		3		0
In paid employment	41 (2.7)		1957 (32.3)	
Retired	1414 (94.3)		2162 (35.7)	
Unemployed/doing voluntary (unpaid) work	44 (2.9)		1934 (32.0)	
Diagnosis subtype (n (%))		0	Not applicable	
Alzheimer’s disease	833 (55.5)			
Vascular dementia	167 (11.1)			
Mixed (Alzheimer’s and vascular)	315 (21.0)			
Frontotemporal dementia	53 (3.5)			
Parkinson’s disease dementia	42 (2.8)			
Dementia with Lewy bodies	52 (3.5)			
Unspecified/other	40 (2.7)			
ACE-III total score, Mean (SD)	69.12 (13.12)	103	Not applicable	
Attention	13.87 (2.99)			
Memory	13.49 (5.38)			
Verbal fluency	6.75 (3.06)			
Language	22.45 (3.63)			
Visuospatial ability	12.51 (3.25)			
Attitudes toward own aging, Mean (SD)	2.36 (1.58)		2.62 (0.79)	
Depression (Depressed; n(%))	441 (30.1)	38	1496 (24.8)	27
Functional ability (Dependent; n(%))	1318 (90.9)	52	463 (7.7)	0
Self-rated health, (n (%))		4		11
Poor	201 (13.4)		120 (2)	
Fair	354 (23.6)		765 (12.7)	
Good	842 (56.2)		3299 (54.6)	
Excellent	101 (6.7)		1858 (30.8)	
Co-morbidity; Mean (SD)	2.91 (1.91)	103	Not applicable	

Note: ACE-III, Addenbrooke’s Cognitive Examination-III

### PROTECT sample characteristics

Of the 14,797 participants that took part in PROTECT 5387 were excluded as they did not complete the ATOA; 3033 were excluded as they did not complete the PROTECT cognitive battery [28] and therefore mild cognitive impairment (MCI) or dementia could not be eliminated; and 178 were excluded as they had missing data in one of the main current study variables. An additional 144 participants (2.4%) were excluded as they either self-reported (*n* = 24) or were judged to have MCI (*n* = 122) based on their scores on two or more cognitive measures included in the PROTECT cognitive battery. Therefore, we included data from 6053 participants, the majority of whom were women (76.2%); the mean age was 66.1 years (range: 51–95), and almost all were white (98.6%). Further characteristics of the study sample are reported in Table 1.

### Differences in levels of ATOA among PwD and people without dementia

Presence of dementia in the unadjusted model was associated with more negative ATOA (*p* <  0.001); thus PwD have more negative ATOA than people without dementia (Table 2). However, the effect size was small and the mean difference in levels of ATOA between PwD and people without dementia was very small (0.2). After adjusting for covariates the model was no longer significant. Being depressed and having poorer self-rated health were significant covariates and were associated with more negative ATOA (Table 2).

**Table 2:** Unadjusted and adjusted associations with dementia diagnosis as the predictor of attitudes toward own aging

	Variables	Regression coefficient (95% CI); *p*- value	Standardized regression coefficient (95% CI)
Unadjusted model	Dementia	− 0.27 (− 0.32 to − 0.21); *<* 0.001	− 0.11 (− 0.13 to − 0.08)
Adjusted model	Dementia	− 0.05 (− 0.12 to 0.01); 0.14	− 0.02 (− 0.05 to 0.01)
	Age	0.00 (0.00 to 0.00); 0.91	− 0.02 (− 0.03 to 0.02)
	Sex	− 0.01 (− 0.05 to 0.04); 0.83	0.00 (− 0.03 to 0.02)
	Employment	− 0.02 (− 0.05 to 0.02); 0.34	−0.01 (− 0.03 to 0.01)
	Education	0.01 (− 0.04 to 0.05); 0.71	0.00 (− 0.02 to 0.03)
	Depression	− 0.45 (− 0.50 to − 0.40); *<* 0.001	− 0.20 (− 0.22 to − 0.17)
	Self-rated health	0.28 (0.25 to 0.31); *<* 0.001	0.22 (0.19 to 0.24)
	Functional ability	− 0.01 (− 0.09 to 0.08); 0.90	0.00 (− 0.02 to 0.02)

### Relationships of ATOA with cognition among PwD

Poorer memory performance, better verbal fluency, and better visuospatial ability predicted more positive ATOA in the unadjusted model; effect sizes were negligible (Table 3). After adjusting for covariates only better visuospatial ability predicted more positive ATOA (Table 3 and supplementary Table 1). In the unadjusted model language ability was associated with ATOA (*p* = 0.044); the size of the association was negligible (Table 3). Neither general cognition nor attention were significantly associated with ATOA at the 5% level (Table 3).

**Table 3:** Unadjusted and adjusted associations with general cognition, memory, verbal fluency, and visuospatial ability, attention, and language as the predictors of attitudes toward own aging

Predictive variables	Unadjusted model Standardized coefficient (95% CI)	Adjusted model* Standardized coefficient (95% CI)
General cognition	0.03 (− 0.02 to 0.09)	0.02 (− 0.03 to 0.06)
Memory	−0.09 (− 0.14 to − 0.04)	−0.03 (− 0.08 to 0.01)
Verbal fluency	0.09 (0.04 to 0.14)	0.02 (− 0.02 to 0.07)
Visuospatial ability	0.15 (0.10 to 0.20)	0.07 (0.02 to 0.12)
Attention	0.02 (− 0.03 to 0.07)	0.01 (− 0.04 to 0.05)
Language	0.05 (0.02 to 0.10)	0.02 (− 0.02 to 0.07)

Note: * Adjusted for age, sex, education, employment, depression, self-rated health, functional ability, and co-morbidity

### Relationship between ATOA and dementia subtypes

Type of dementia predicted a significant amount of variability in ATOA before controlling for covariates (Table 4). People with Parkinson’s disease dementia and dementia with Lewy bodies reported most negative ATOA (Table 5). Type of dementia did not predict a significant amount of variability in ATOA after controlling for covariates (Table 4). Depression, self-rated health, and self-reported visual acuity were significant covariates.

**Table 4:** Unadjusted and adjusted associations with dementia subtypes as the predictor of attitudes toward own aging

	Variables	Regression coefficient (95% CI); *p*- value	Standardized regression coefficients
Unadjusted model	Dementia subtypes:		
	Alzheimer’s disease	Reference group	Reference group
	Vascular dementia	−0.30 (− 0.55 to − 0.04); 0.025	−0.06
	Mixed (Alzheimer’s and vascular)	−0.28 (− 0.48 to − 0.08); 0.006	−0.07
	Frontotemporal dementia	−0.17 (− 0.60 to 0.26); 0.439	− 0.02
	Parkinson’s disease dementia	−1.32 (− 1.80 to − 0.83); < 0.001	−0.14
	Dementia with Lewy bodies	−0.89 (− 1.33 to − 0.46); < 0.001	−0.10
	Unspecified/other	−0.25 (− 0.75 to 0.24); 0.313	−0.03
Adjusted model	Dementia subtypes:		
	Alzheimer’s disease	Reference group	Reference group
	Vascular dementia	0.02 (−0.23 to 0.26); 0.879	0.00
	Mixed (Alzheimer’s and vascular)	−0.08 (− 0.27 to 0.11); 0.393	−0.02
	Frontotemporal dementia	−0.22 (− 0.61 to 0.16); 0.258	−0.02
	Parkinson’s disease dementia	− 0.36 (− 0.82 to 0.10); 0.122	−0.04
	Dementia with Lewy bodies	−0.13 (− 0.54 to 0.28); 0.542	−0.01
	Unspecified/other	0.11 (−0.36 to 0.58); 0.649	0.01
	Covariates:		
	Age	−0.00 (−0.01 to 0.01); 0.417	− 0.02
	Sex	−0.04 (− 0.18 to 0.11); 0.596	−0.01
	Education	−0.05 (− 0.23 to 0.13); 0.555	−0.01
	Employment	−0.09 (− 0.39 to 0.20); 0.538	−0.01
	Depression	−1.46 (−1.64 to − 1.29); *<* 0.001	− 0.42
	Self-rated health	0.37 (0.26 to 0.50); < 0.001	0.19
	Functional ability	−0.27 (− 0.63 to 0.10); *<* 0.001	−0.03
	Co-morbidity	−0.01 (− 0.05 to 0.03); 0.504	−0.02
	Visual acuity	−0.16 (− 0.24 to − 0.09); *<* 0.001	−0.11

**Table 5:** Means, standard deviation, and comparison of levels on the Attitudes Toward Own Aging scale across seven dementia subtypes

Analysis of variance between dementia subtypes and attitudes toward own aging					
Dementia subtypes	Attitudes toward own aging Mean (SD)	Contrast (95% CI)	Standard Error	Eta-squared	*F*-statistic (d*f*); *p*
Alzheimer’s disease	2.53 (1.58)	(reference)	(reference)	0.03	7.79 (6); < .001
Vascular dementia	2.23 (1.70)	−0.30 (− 0.64 to 0.05)	0.13		
Mixed (Alzheimer’s and vascular)	2.25 (1.48)	−0.28 (− 0.55 to − 0.01)	0.10		
Frontotemporal dementia	2.35 (1.51)	−0.17 (− 0.75 to 0.41)	0.22		
Parkinson’s disease dementia	1.21 (1.20)	−1.32 (−1.97 to −0.66)	0.25		
Dementia with Lewy Bodies	1.63 (1.39)	−0.89 (−1.48 to − 0.31)	0.22		
Unspecified/other	2.28 (1.71)	−0.25 (− 0.92 to 0.41)	0.25		

### Relationship between visuospatial ability and ATOA

As better visuospatial ability predicted more positive ATOA, we explored whether self-reported visual acuity explains variability in levels of ATOA. Before and after controlling for covariates, individuals with poorer self-reported visual acuity reported more negative ATOA than those with better self-reported visual acuity; the effect size was small (Table 6).

**Table 6:** Unadjusted and adjusted associations with visual acuity as the predictor of attitudes toward own aging

	Variables	Regression coefficient (95% CI); *p*- value	Standardized regression coefficients
Unadjusted model	Visual acuity Excellent		
	(Reference)	−0.41 (− 0.69 to − 0.12); 0.005	−0.11
	Very good	−0.80 (−1.07 to − 0.53); *<* 0.001	−0.25
	Good	−1.40 (− 1.70 to − 1.09); *<* 0.001	−0.34
	Fair	− 1.70 (−2.09 to − 1.32); *<* 0.001	− 0.26
	Poor		
Adjusted model	Visual acuity Excellent		
	(Reference)	−0.28 (−0.54 to − 0.01); 0.039	−0.08
	Very good	−0.40 (− 0.65 to − 0.15); 0.002	−0.12
	Good	−0.61 (− 0.91 to − 0.32); < 0.001	−0.15
	Fair	−0.62 (−1.0 to − 0.24); 0.001	−0.09
	Poor		
	Age	−0.00 (− 0.01 to 0.01); 0.422	−0.02
	Sex	−0.03 (− 0.17 to 0.11); 0.682	−0.01
	Education	−0.05 (− 0.43 to 0.13); 0.552	−0.01
	Employment	−0.11 (− 0.41 to 0.19); 0.478	−0.02
	Depression	−1.47 (−1.65 to − 1.30); *<* 0.001	− 0.42
	Self-rated health	0.37 (0.27 to 0.48); *<* 0.001	0.19
	Functional ability	−0.28 (− 0.65 to 0.08); 0.127	−0.03
	Co-morbidity	−0.01 (− 0.05 to 0.03); 0.503	−0.02

## Discussion

This study showed no difference in levels of ATOA between PwD and people without dementia. Among PwD those with more extensive cognitive impairment do not have more negative ATOA than those with better cognition. These results are promising as positive ATOA is a psychological resource that helps PwD to live well with dementia [19, 20]. Among dementia subtypes, people with Parkinson’s disease dementia and dementia with Lewy bodies report the most negative ATOA; hence they may lack a potentially important psychological resource.

Consistently with previous studies we found very small differences in levels of ATOA between PwD and people without dementia [19, 20]; however, these previous studies were limited in that they did not control for potentially important covariates. After controlling for covariates, we demonstrated that having a dementia diagnosis was no longer associated with more negative ATOA. Being depressed and having poorer self-rated health were significant covariates associated with more negative ATOA among PwD and people without dementia. Hence the slightly more negative ATOA reported by PwD may be due to them being more likely to experience depression and poor self-rated health [16, 17] than people without dementia. Based on the small mean difference in ATOA our findings suggest no difference in levels of ATOA between PwD and people without dementia and any differences are likely due to other factors including self-rated depression, reduced visual acuity and/or health.

Only one study with a small sample size has so far explored the association of general cognition with ATOA in PwD, and found no association [20]. Consistent with this study, we found no association in PwD between general cognition and ATOA. The lack of an association is interesting as despite poorer cognitive performance being associated with more negative ATOA and increased likelihood of developing dementia among middle-aged and older individuals [7–10], in people with established dementia degree of cognitive impairment does not appear to be related to ATOA. The cognitive failures that middle-aged and older individuals without a diagnosis of dementia experience may be unexpected and hence have a negative impact on self-perceptions including ATOA. Receiving a diagnosis of dementia may lead to the internalization of negative attributes about the self [42] including negative ATOA, but the subsequent cognitive decline that comes with the progression of the disease may not further exacerbate negative ATOA.

We explored the association of ATOA with five cognitive subdomains among PwD. Poorer executive function and poorer verbal ability have been associated with more negative ATOA among middle-aged and older individuals [7]. Among PwD, we found that those with poorer visuospatial ability had more negative ATOA. Importantly, we found a small association for self-rated poorer visual acuity with more negative ATOA in PwD, suggesting that age-related changes in vision may negatively impact both on ATOA and visuospatial ability. Further studies could focus on the association between ATOA, poor eyesight and visuospatial ability with a more comprehensive visuospatial assessment. We also found that PwD with better memory and poorer verbal fluency had more negative ATOA, though effects were not statistically significant after controlling for covariates. Depression, poorer self-rated health, and lower functional ability predicted more negative ATOA. For PwD with better memory who are at an early stage of the illness, more negative ATOA, psychological well-being, and self-rated health could be due to having recently received a diagnosis of dementia [42]. The negative ATOA reported by PwD with poor verbal fluency may be due to these individuals having difficulty communicating basic needs or interacting with family members on a day-to-day basis [43].

Finally, the finding that people with Parkinson’s disease dementia and dementia with Lewy bodies have more negative ATOA than people with other types of dementia extends previous research where people with these two dementia subtypes reported poorer psychological health and quality of life [44, 45]. The more negative ATOA found in people with these two dementia subtypes may be due to the movement disorders and visuospatial abnormalities associated with their Parkinsonian pathology. Indeed, restricted movement can limit daily activities [45] which may negatively affect ATOA. Differences across dementia subtypes were attenuated after controlling for covariates, with depression, self-rated health, and self-reported visual acuity being significant covariates. This further suggests that the motor and visual impairments [45, 46] that characterize people with Parkinson’s disease dementia and dementia with Lewy bodies may negatively impact ATOA in these conditions. It may be that motor and visual impairments are more salient than cognitive impairment in terms of the impact on ATOA. Future studies could explore, in people with Parkinson’s disease without dementia, whether greater motor and visual impairments are associated with poorer ATOA.

### Strengths and limitations

Combining participants from the IDEAL and PROTECT studies is both a strength and limitation of this study. The two cohorts recruited and assessed participants in different ways which may have influenced participants’ answers. In addition participants in IDEAL were generally older and less educated, and the gender-balance in the sample was more even compared to PROTECT. Also, IDEAL included fewer participants that were still employed than participants in PROTECT. These factors were controlled for in the analyses; therefore the statistically significant though very small difference in levels of ATOA between PwD and people without dementia was unlikely to be due to the demographic characteristics of the samples. Participants in both cohorts were mainly white; as there are cultural differences in ATOA, generalization of results to other ethnic groups should be exercised with caution [47]. Harmonizing different measures employed between the IDEAL and PROTECT studies to assess the same behavioral characteristics may have generated some bias, although harmonizing in this way between different studies is well-established [48].

Due to the breadth of the IDEAL and PROTECT studies, only brief measures could be used. This study did not use a domain-specific measure of ATOA; this is a limitation as levels of ATOA may differ between PwD and people without dementia depending on the domain studied [19, 20, 49]. Visual acuity was self-rated by participants; this is a potential limitation as the association we found between poorer self-reported visual acuity and more negative ATOA may be due to people with more negative ATOA expecting a decline in their eyesight as part of getting older. Future studies could explore whether the association of ATOA with visual acuity is confirmed when using objective assessments of visual impairment. Finally, the current study is based on cross-sectional analyses. Those with more negative ATOA may show greater cognitive decline over time; therefore the association between cognition and ATOA among PwD may become significant longitudinally. Analyses to investigate this will be conducted once longitudinal data are available.

A strength of the study is the use of two large cohorts that facilitated exploring differences in levels of ATOA among PwD and people without dementia, particularly in some of the rarer dementia subtypes. Another strength of the study is that IDEAL included people with any type of dementia in roughly the same proportions diagnosed in memory clinics, and people from a range of economic backgrounds [50]. A final strength is that using the ACE-III made it possible to explore in PwD the associations of ATOA with five cognitive subdomains.

## Conclusion

This study showed that while PwD report slightly more negative ATOA than people without dementia, this effect disappears after controlling for depression and self-rated health. In PwD ATOA are not affected by the degree of cognitive impairment. Finally, people with a diagnosis of Parkinson’s disease dementia or dementia with Lewy bodies expressed more negative ATOA compared to people with other dementia diagnoses; this may be due to the motor and visual impairments that they experience. Further investigation of the associations of more negative ATOA with poorer visuospatial ability and greater visual impairment in PwD is needed as visual impairments may be a potential risk factor for negative ATOA. Overall, even though levels of ATOA do not differ between people with and without dementia, among PwD, those with Parkinson’s disease dementia or dementia with Lewy bodies may be at highest risk of experiencing negative ATOA.

## Supplementary Information

**Additional file 1: Supplementary Table 1**. Regression models with attitudes toward own aging as the predictor of memory, verbal fluency, and visuospatial ability.

## Data Availability

IDEAL data were deposited with the UK data archive in April 2020 and will be available to access from April 2023. Details of how the data can be accessed after that date can be found here: http://reshare.ukdataservice.ac.uk/854293/. PROTECT data are available to investigators outside the PROTECT team after request and approval by the PROTECT Steering Committee.

## Abbreviations

PwD: People with dementia
ACE-III: Addenbrooke’s Cognitive Examination-III
ATOA: Attitudes toward own aging
CRN: Clinical Research Network
FAQ: Functional Activities Questionnaire
IADL: Activities of Daily Living Scale
IDEAL: Improving the experience of Dementia and Enhancing Active Life
M: Mean
MCI: Mild cognitive impairment
MMSE: Mini-Mental State Examination
NHS: National Health Service
PHQ: Patient Health Questionnaire
PROTECT: Platform for Research Online to investigate Genetics and Cognition in Aging
SD: Standard Deviation
η^2^/pη^2^: Eta-squared and partial eta-squared

## References

[1] Alzheimer's Disease International (2019). World Alzheimer report 2019 attitudes to dementia.

[2] Lawton MP (1975). The Philadelphia geriatric center morale scale: a revision. J Gerontol.

[3] Kite ME, Stockdale GD, Whitley BE, Johnson BT (2005). Attitudes toward younger and older adults: an updated meta-analytic review. J Soc Issues.

[4] Levy BR, Zonderman AB, Slade MD, Ferrucci L (2009). Age stereotypes held earlier in life predict cardiovascular events in later life. Psychol Sci.

[5] Seidler AL, Wolff JK (2017). Bidirectional associations between self-perceptions of aging and processing speed across 3 years. J Gerontopsychol Geriatric Psychiatry.

[6] Slot RER, Sikkes SAM, Berkhof J, Brodaty H, Buckley R, Cavedo E (2019). Subjective cognitive decline and rates of incident Alzheimer's disease and non-Alzheimer's disease dementia. Alzheimers Dement.

[7] Siebert JS, Wahl H-W, Schröder J (2016). The role of attitude toward own aging for fluid and crystallized functioning: 12-year evidence from the ILSE study. J Gerontol Ser B Psychol Sci Soc Sci.

[8] Levy BR, Slade MD, Pietrzak RH, Ferrucci L (2018). Positive age beliefs protect against dementia even among elders with high-risk gene. PLoS One.

[9] Levy BR, Ferrucci L, Zonderman AB, Slade MD, Troncoso J, Resnick SM (2016). A culture-brain link: negative age stereotypes predict Alzheimer's disease biomarkers. Psychol Aging.

[10] Siebert JS, Wahl H-W, Degen C, Schröder J (2018). Attitude toward own aging as a risk factor for cognitive disorder in old age: 12-year evidence from the ILSE study. Psychol Aging.

[11] Hess TM, Birren J, Schaie KW (2006). Attitudes toward aging and their effects on behavior. Handbook of the psychology of aging.

[12] Anstey KJ (2013). Optimizing cognitive development over the life course and preventing cognitive decline: introducing the cognitive health environment life course model (CHELM). Int J Behav Dev.

[13] Martyr A, Clare L. Executive function and activities of daily living in Alzheimer’s disease: a correlational meta-analysis. Dement Geriatr Cogn Disord 2012;33(2–3):189–203. 10.1159/000338233 22572810

[14] American Psychiatric Association (2013). Diagnostic and statistical manual of mental disorders (DSM-5®).

[15] Marshall GA, Rentz DM, Frey MT, Locascio JJ, Johnson KA, Sperling RA (2011). Executive function and instrumental activities of daily living in mild cognitive impairment and Alzheimer’s disease. Alzheimers Dement.

[16] Bunn F, Burn A-M, Goodman C, Rait G, Norton S, Robinson L (2014). Comorbidity and dementia: a scoping review of the literature. BMC Med.

[17] Nelis SM, Wu YT, Matthews FE, Martyr A, Quinn C, Rippon I (2019). The impact of co-morbidity on the quality of life of people with dementia: findings from the IDEAL study. Age Ageing.

[18] Martyr A, Nelis SM, Quinn C, Wu Y-T, Lamont RA, Henderson C (2018). Living well with dementia: a systematic review and correlational meta-analysis of factors associated with quality of life, well-being and life satisfaction in people with dementia. Psychol Med.

[19] Kisvetrová H, Herzig R, Bretšnajdrová M, Tomanová J, Langová K, Školoudík D. Predictors of quality of life and attitude to ageing in older adults with and without dementia. Aging Ment Health. 2019. 10.1080/13607863.2019.1705758 31870177

[20] Trigg R, Watts S, Jones R, Tod A, Elliman R (2012). Self-reported quality of life ratings of people with dementia: the role of attitudes to aging. Int Psychogeriatr.

[21] Sargent-Cox KA, Anstey KJ, Luszcz MA (2012). The relationship between change in self-perceptions of aging and physical functioning in older adults. Psychol Aging.

[22] Kotter-Grühn D, Kleinspehn-Ammerlahn A, Gerstorf D, Smith J (2009). Self-perceptions of aging predict mortality and change with approaching death: 16-year longitudinal results from the Berlin aging study. Psychol Aging.

[23] Institute of Medicine (2012). Living well with chronic illness: a call for public health action.

[24] Clare L, Wu YT, Jones IR, Victor CR, Nelis SM, Martyr A (2019). A comprehensive model of factors associated with subjective perceptions of "living well" with dementia: findings from the IDEAL study. Alzheimer Dis Assoc Disord.

[25] Clare L, Nelis SM, Quinn C, Martyr A, Henderson C, Hindle JV (2014). Improving the experience of dementia and enhancing active life-living well with dementia: study protocol for the IDEAL study. Health Qual Life Outcomes.

[26] Silarova B, Nelis SM, Ashworth RM, Ballard C, Bieńkiewicz M, Henderson C (2018). Protocol for the IDEAL-2 longitudinal study: following the experiences of people with dementia and their primary carers to understand what contributes to living well with dementia and enhances active life. BMC Public Health.

[27] Sabatini S, Ukoumunne OC, Ballard C, Brothers AF, Kaspar R, Collins R, et al. International relevance of two measures of awareness of age-related change (AARC). BMC Geriatr. 2020;20(1). 10.1186/s12877-020-01767-6 PMC7507664 32957978

[28] Corbett A, Owen A, Hampshire A, Grahn J, Stenton R, Dajani S (2015). The effect of an online cognitive training package in healthy older adults: an online randomized controlled trial. J Am Med Dir Assoc.

[29] Kaspar R, Gabrian M, Brothers AF, Wahl H-W, Diehl MK (2019). Measuring awareness of age-related change: development of a 10-item short form for use in large-scale surveys. The Gerontologist.

[30] Hsieh S, Schubert S, Hoon C, Mioshi E, Hodges JR (2013). Validation of the Addenbrooke's cognitive examination III in frontotemporal dementia and Alzheimer's disease. Dement Geriatr Cogn Disord.

[31] Almeida OP, Almeida SA (1999). Short versions of the geriatric depression scale: a study of their validity for the diagnosis of a major depressive episode according to ICD-10 and DSM-IV. Int J Geriatr Psychiatry.

[32] Bowling A (2005). Just one question: if one question works, why ask several?. J Epidemiol Community Health.

[33] Charlson ME, Charlson RE, Peterson JC, Marinopoulos SS, Briggs WM, Hollenberg JP (2008). The Charlson comorbidity index is adapted to predict costs of chronic disease in primary care patients. J Clin Epidemiol.

[34] Charlson ME, Pompei P, Ales KL, MacKenzie CR (1987). A new method of classifying prognostic comorbidity in longitudinal studies: development and validation. J Chronic Dis.

[35] English Longitudinal Study of Ageing (2018). English Longitudinal Study of Ageing (ELSA).

[36] Ware JE, Sherbourne CD (1992). The MOS 36-item short-form health survey (SF-36). Conceptual framework and item selection. Med Care.

[37] Lawton MP, Brody EM (1969). Assessment of oder people: self-maintaining and instrumental activities of daily living. The Gerontologist.

[38] Kroenke K, Spitzer RL, Williams JB (2001). The PHQ-9: validity of a brief depression severity measure. J Gen Intern Med.

[39] Pfeffer RI, Kurosaki TT, Harrah CH, Chance JM, Filos S (1982). Measurement of functional activities in older adults in the community. J Gerontol.

[40] Martyr A, Nelis SM, Quinn C, Rusted JM, Morris RG, Clare L (2019). The relationship between perceived functional difficulties and the ability to live well with mild-to-moderate dementia: findings from the IDEAL programme. Int J Geriatr Psychiatry.

[41] Cohen J (1988). Statistical power analysis for the behavioral sciences.

[42] Clare L, Martyr A, Morris RG, Tippett LJ (2020). Discontinuity in the subjective experience of self among people with mild-to-moderate dementia is associated with poorer psychological health: findings from the IDEAL cohort. J Alzheimers Dis.

[43] Alsawy S, Mansell W, McEvoy P, Tai S (2017). What is good communication for people living with dementia?. A mixed-methods systematic review Int Psychogeriatr.

[44] Wu Y-T, Clare L, Hindle JV, Nelis SM, Martyr A, Matthews FE (2018). Dementia subtype and living well: results from the improving the experience of dementia and enhancing active life (IDEAL) study. BMC Med.

[45] Berganzo K, Tijero B, Gonzalez-Eizaguirre A, Somme J, Lezcano E, Gabilondo I (2016). Motor and non-motor symptoms of Parkinson's disease and their impact on quality of life and on different clinical subgroups. Neurologia..

[46] Moschos MM, Tagaris G, Markopoulos L, Margetis L, Tsapakis S, Kanakis M (2010). Morphologic changes and functional retinal impairment in patients with Parkinson disease without wisual loss. Eur J Ophthalmol.

[47] Westerhof GJ, Barrett AE (2005). Age identity and subjective well-being: a comparison of the United States and Germany. J Gerontol Ser B Psychol Sci Soc Sci.

[48] Khachaturian AS, Meranus DH, Kukull WA, Khachaturian ZS (2013). Big data, aging, and dementia: pathways for international harmonization on data sharing. Alzheimers Dement.

[49] Sabatini S, Silarova B, Martyr A, Collins R, Ballard C, Anstey KJ (2020). Associations of awareness of age-related change with emotional and physical well-being: a systematic review and meta-analysis. The Gerontologist.

[50] Wu Y-T, Clare L, Jones IR, Martyr A, Nelis SM, Quinn C (2018). Inequalities in living well with dementia—the impact of deprivation on well-being, quality of life and life satisfaction: results from the improving the experience of dementia and enhancing active life study. Int J Geriatr Psychiatry.

