# Supplementary material for: Attitudes toward own aging and cognition among individuals living with and without dementia: findings from the IDEAL programme and the PROTECT study
Source: BMC Geriatr. 2022 Aug 4;22:641. doi: 10.1186/s12877-022-03336-5 (PMC9351129; doi:10.1186/s12877-022-03336-5)
Supplement: Supplementary file 1 — Additional file 1: Supplementary Table 1. Regression models with attitudes toward own aging as the predictor of memory, verbal fluency, and visuospatial ability. [file 12877_2022_3336_MOESM1_ESM.docx]

**Supplementary Table 1. Regression models with attitudes toward own aging as the predictor of memory, verbal fluency, and visuospatial ability**

| Memory as predictor of attitudes toward own aging | | | |
| --- | --- | --- | --- |
|  | Variables | Regression coefficient (95% CI); *p*- value | Standardized regression coefficient (95% CI) |
| Unadjusted model | Memory | -0.03 (-0.04 to -0.01); 0.001 | -0.09 (-0.14 to -0.04) |
| Adjusted model | Memory | -0.01 (-0.02 to 0.00); 0.154 | -0.03 (-0.08 to 0.01) |
|  | Age | -0.00 (-0.01 to 0.01); 0.425 | -0.02 (-0.07 to 0.03) |
|  | Sex | -0.05 (-0.20 to 0.10); 0.520 | -0.02 (-0.06 to 0.03) |
|  | Education | 0.01 (-0.18 to 0.19); 0.946 | 0.00 (-0.04 to 0.05) |
|  | Employment | -0.12 (-0.45 to 0.18); 0.446 | -0.02 (-0.07 to 0.03) |
|  | Depression | -1.55 (-1.72 to -1.37); *<* 0.001 | -0.44 (-0.48 to -0.40) |
|  | Self-rated health | 0.44 ( 0.34 to 0.54); *<* 0.001 | 0.22 ( 0.17 to 0.27) |
|  | Functional ability | -0.44 (-0.84 to -0.05); 0.027 | -0.05 (-0.10 to -0.01) |
|  | Co-morbidity | -0.01 (-0.05 to 0.03); 0.723 | -0.01 (-0.06 to 0.04) |
| Verbal fluency as predictor of attitudes toward own aging | | | |
|  | Variables | Regression coefficient (95% CI) | Standardized regression coefficient (95% CI) |
| Unadjusted model | Verbal fluency | 0.04 ( 0.02 to 0.07); 0.001 | 0.09 ( 0.04 to 0.14) |
| Adjusted model | Verbal fluency | 0.01 (-0.01 to 0.04); 0.340 | 0.02 (-0.02 to 0.07) |
|  | Age | -0.00 (-0.01 to 0.01); 0.599 | -0.01 (-0.06 to 0.03) |
|  | Sex | -0.04 (-0.18 to 0.11); 0.619 | -0.01 (-0.06 to 0.03) |
|  | Education | -0.04 (-0.22 to 0.15); 0.689 | -0.01 (-0.05 to 0.04) |
|  | Employment | -0.13 (-0.44 to 0.19); 0.439 | -0.02 (-0.06 to 0.03) |
|  | Depression | -1.53 (-1.71 to -1.36); *<* 0.001 | -0.44 (-0.48 to -0.39) |
|  | Self-rated health | 0.44 ( 0.34 to 0.54); *<* 0.001 | 0.22 ( 0.18 to 0.27) |
|  | Functional ability | -0.36 (-0.74 to 0.03); 0.070 | -0.04 (-0.09 to 0.00) |
|  | Co-morbidity | -0.01 (-0.05 to 0.03); 0.483 | -0.02 (-0.06 to 0.03) |
| Visuospatial ability as predictor of attitudes toward own aging | | | |
|  | Variables | Regression coefficient (95% CI) | Standardized regression coefficient (95% CI) |
| Unadjusted model | Visuospatial ability | 0.07 ( 0.05 to 0.10); *<* 0.001 | 0.15 ( 0.10 to 0.20) |
| Adjusted model | Visuospatial ability | 0.03 ( 0.01 to 0.06); 0.004 | 0.07 ( 0.02 to 0.12) |
|  | Age | -0.00 (-0.01 to 0.01); 0.389 | -0.02 (-0.07 to 0.03) |
|  | Sex | -0.03 (-0.18 to 0.11); 0.667 | -0.01 (-0.06 to 0.04) |
|  | Education | -0.03 (-0.21 to 0.15); 0.737 | -0.01 (-0.05 to 0.04) |
|  | Employment | -0.17 (-0.48 to 0.15); 0.354 | -0.02 (-0.07 to 0.02) |
|  | Depression | -1.53 (-1.70 to -1.35); *<* 0.001 | -0.43 (-0.48 to -0.39) |
|  | Self-rated health | 0.43 ( 0.33 to 0.53); *<* 0.001 | 0.22 ( 0.17 to 0.27) |
|  | Functional ability | -0.28 (-0.67 to 0.10); 0.150 | -0.03 (-0.08 to 0.01) |
|  | Co-morbidity | -0.02 (-0.06 to 0.02); 0.442 | -0.02 (-0.07 to 0.03) |
